# Managers in the publicly funded health services in China - characteristics and responsibilities

**DOI:** 10.1186/s12913-020-05577-9

**Published:** 2020-08-06

**Authors:** Zhanming Liang, Felicity C. Blackstock, Peter Howard, Chaojie Liu, Geoffrey Leggat, Hongkun Ma, Zhijun Zhang, Timothy Bartram

**Affiliations:** 1 School of Psychology and Public Health, College of Science, Health and Engineering, La Trobe University, Bundoora, Victoria Australia; 2 School of Science and Health, Western Sydney University, Campbelltown, New South Wales Australia; 3 Dongbei Agriculture University, Harbin, China; 4 Puyuhuang Community Health Centre, Beijing, China; 5 School of Management, College of Business, RMIT University, Melbourne, Victoria Australia

**Keywords:** Chinese public health system, Health service managers, Educational background, Training needs, Managerial tasks

## Abstract

**Background:**

Health service managers are integral to supporting the effective and efficient delivery of services. Understanding their competencies is essential to support reform and improvement of healthcare provision in China. This paper examines the characteristics and educational background of senior managers working in the community health and hospital sectors in China. We also examine their levels of commitment to continued professional development and continuous education.

**Methods:**

A self-administered paper-based questionnaire was administered to 477 level I, II and III managers in community health services and public hospitals in China. The response rate was over 80%.

**Results:**

Findings demonstrate significant differences in terms of educational background and commitment to ongoing professional development between the managers in China across levels of management, and between the community and hospital sectors. Hospital managers tend to be older; hospital managers at higher management levels are predominantly male but predominantly female in the community health services. A greater proportion of hospital managers have postgraduate qualifications. In addition, the participants identified specific management tasks that they considered important.

**Conclusions:**

This is the first large scale study examining the educational background and commitment to professional development of senior health service managers in China. This study determined that there are differences between the demographics of managers in China across levels of management, but more importantly between the CHC and the hospital sectors. The identification of important managerial tasks will facilitate the development of appropriate education and training for Chinese healthcare managers. All sectors and levels reported the need for informal education focussed on the core roles of developing organisation image and public relations, improving quality and safety of service provision and provision of leadership. Further research to explore the underlying reasons for the above differences is needed to design appropriate professional development for China’s health services managers. In addition, the importance of managerial tasks across sectors and management levels requires further investigation.

## Background

Increasing health expenditure and changing healthcare needs have led to significant changes in health service provision and operational models worldwide since the early 1980s [1]. Studies have examined how constant changes in the healthcare system effect healthcare managers and their key role in leading and managing the reform agenda and ensuring the effectiveness and efficiency of health service provision [2]. However, evidence on ways of equipping health service managers in terms of improving their management competence, and thereby management outcomes of better service provision, is still far from adequate [3–5]. Despite increasing emphasis on training and development of health service managers, there is a dearth of literature on organisational and governmental strategies to address the lack of training and skill development for health professionals taking on management roles in developing countries [6–8]. The paucity of literature illustrates a lack of understanding of professional development opportunities and needs, and suggests there may be inadequate preparation of clinicians (e.g., medical doctors, nurses) for management roles [9, 10]. To date no empirical evidence has identified the training and competency development needs of managers working in the Chinese healthcare system.

### Reform of the Chinese health system

The Chinese healthcare system is facing unique challenges. The adoption of the cost-recovery health service funding model via revenue generated from service charges has resulted in overprovision of services and the wasteful use of resources in the Chinese health system, which further reduces the equality and affordability of basic healthcare service [11–13]. Although the majority of the health services in China are publicly owned, under such a financially driven funding model, they could be considered ‘private, for-profit’ in terms of management behaviou r[14].^(p614)^ This financially driven system has inevitably further increased the burden to the national health insurance scheme, and high out of pocket expenses for patients [13, 15, 16]. The result is that the Chinese health services are now less affordable for both the individual and society as a whole [17] and with increasing inequality of health service provision and poorer health outcomes.

Although improvements have been achieved in the past decade by reaching 96% of the population with social insurance schemes [18], the out of pocket expenses for healthcare services is still comparatively high at about 8.5% of total household expenditure in China [17] as of 2012 in comparison with the 3.2% in Australia [19]. In 2014, out of pocket expenses for healthcare services was around 36% of the total health expenditure in China in comparison with the 18.15% world average; 18.8% in Australia, 7.9% in Thailand, 9.7% in UK and 11% in the US [20, 21]. Apparently the challenge of affordable and efficient health care for all in China is yet to be addressed.

In these contexts, reforms in the Chinese health system were announced in 2009 with an agenda emphasising building a strong delivery system based on primary health care, anchored in community health centres in cities and township health centres in rural areas [22]. The reforms also focused on the improvement of the administration and governance of public hospitals focussing on hospital internal management, governance, compensation systems, supervising mechanisms and improving medical service quality [23].

In 2012, the total population in China was slightly more than 1.354 billion [24]. There were 947,873 healthcare organisations in China, which included 23,170 hospitals, 912,620 primary health care institutions, of which 33,562 are community health centres primarily located in the urban areas, and 12,083 public health agencies and other healthcare organisations [21]. Amongst the hospitals, roughly half of them are government funded public hospitals and the other half are private organisations independently funded, but hospital beds are still predominately located in public hospitals. In urban China, most of the outpatient visits to general practitioners and specialists currently still take place in hospital settings, although in some major cities, such visits are now strongly encouraged to the community health centres [21].

### Competency and skill challenges to health service managers

Historically, hospitals were the major health service providers in China before the Community Health Centres (CHC) were initiated in the 1990s as an effort to revitalising primary healthcare. The reform agenda of strengthening primary healthcare provisions further reinforced the importance of non-hospital service provision. This shift of health service delivery model required a large workforce to meet the demand of primary healthcare service provision.

Senior managers in both hospitals and the CHCs play critical roles in ensuring the success of the implementation of any reform strategies and the effective and efficient service provision in the sector. As discussed earlier, the revenue generation hospital funding model and increasing competition between the public and private providers inevitably put pressure on hospital managers to balance over-service provision and service efficiency. Such pressure may not be experienced by managers working in CHCs as they are fully funded by governments; revenues collected by CHCs from user charges are surrendered to health authorities in exchange for governmental full budget support [21]. However, the historically lower training requirements for health professionals working in CHCs made improving service quality and efficiency in the CHCs difficult – a challenge that may be unique to CHC managers.

Moreover, the government has maintained strict control over the appointment of senior management positions, which are generally promoted internally based on seniority without adequate management skills or systematic training. The lack of systematic training of professionals working in CHCs may mean that managers promoted internally maybe even less prepared and more disadvantaged than those working in the hospital system [25]. Managers of public hospitals and CHCs are also given an equivalent official rank similar to those who work for governmental agencies. The ranking of CHC managers is generally lower than that of hospital managers [25, 26]. Therefore, the development requirements of the management workforce in hospitals and CHCs may be different. In addition, the Ministry of Health requires all health services managers to receive management training, but the implementation is challenging because i) almost all managers are promoted from clinicians who have obtained a formal medical qualification (e.g. Masters or doctoral degree) and ii) postgraduate training courses are usually research focussed.

In China, vast majority of the postgraduate level training is research based, very few tertiary educational providers are able to offer management focussed and practice-based training for health managers [25]. Since health service managers are very commonly selected amongst clinicians, they are more likely to choose clinically related discipline as the focus of the postgraduate study to advance their clinical practice and competence instead of management competence. Informal training is organised by healthcare organisations on an ad hoc basis usually offered by pharmaceutical companies and is clinically based.

In reality, clinicians often take up management roles without a job description with unclear roles and competency requirements. Studies examining the management responsibilities and management requirements of current health service managers in China are still lacking. Furthermore, studies of human resource management (HRM) competency in the Chinese healthcare context are very limited. In a recent systematic review [3], identified 265 papers focusing on research in HRM practices in China and published in English over the past 10 years. Amongst them, only 21 papers are in the area of training and development, organisational learning, leadership, management development or entrepreneurship for HRM managers, and none of them focused on the HRM in the health care sector. These findings are in sharp contrast to the growing evidence in public health care sector management in the UK, US, Canada and Australia since the 1990s [3]. The paucity of literature illustrates a lack of understanding of professional development opportunities and needs, and suggests there may be inadequate preparation of clinicians (e.g., medical doctors, nursing) for management roles [9, 10]. To date no empirical evidence has identified the training and competency development needs of managers working in the Chinese healthcare system. In this paper, we take up this challenge.

Our study aims to develop an understanding of the perceived management roles and professional development needs of the senior health service management workforce in China. It will develop our understanding of the demographic and educational background of the senior Chinese managers working in the public hospitals and community health centres. This paper addresses the following questions:

1. What are the characteristics and educational background of senior managers working in the community health and hospital sectors in China?
2. What are the levels of commitment to continued professional development and continuous education of senior managers working in community health and hospitals in China?
3. What are the core responsibilities for senior managers working in the community health and hospital sectors in China?

A second paper is in preparation. This will report the results of a self-assessment of the competency levels (six competency groups with 79 behavioural items) by the same target population, and their predictors.

## Methods

The study design was a descriptive, cross-sectional, paper-based survey conducted with senior managers (from the top three management levels) working in all 13 CHCs in the Fengtai District, Beijing and all four level 3 teaching hospitals associated with the Harbin Medical University in Harbin.

The four teaching hospitals in Harbin are tertiary hospitals, including three comprehensive hospitals each with over 4500 beds and one cancer hospital with 734 beds. The 13 CHCs are governed by the Fengtai District Community Health Management Committee. A typical CHC usually serves a community of about 100,000 population. They provide a wide range of community health care covering prevention, maternal and child health care, aged care, medical treatments, rehabilitation, traditional Chinese medicine, family planning and public health services. Some CHCs may also have limited hospital beds.

Senior managers for the study are from organisational level, functional division level, and clinical department level

- Level I: Directors, Associate Directors and Secretary of Party Committee;
- Level II: Heads and Deputy Heads of Functional Departments
- Level III: Heads and Deputy Heads of Clinical Divisions

### Target population and sampling

For community health centres, questionnaires were sent to all managers in the targeted three management levels working in the 13 community health centres in the Fengtai District, Beijing (Level I = 31; Level II = 110, and Level III = 142) by the Director of Human Resources who are not part of the research team at the management team meetings. Management meetings are compulsory for all managers attend. This gives a combined sample size of 283 (representing 100% of the Level I, II and III managers in these 13 community health centres).

There were five Level I, 60 Level II and 100 Level III managers in each of four targeted hospitals in Harbin. Random sampling methods were used to target Level II and III managers. Questionnaires were distributed to all five Directors and Deputy Directors and randomly distributed to 25 Head and Deputy Heads of functional departments and 35 Head and Deputy Heads of Clinical divisions at each of the teaching hospitals at a management meeting. This gave a combined target sample size of 260 (representing the Level I managers and 37.5% of the Level II and III managers in the four hospitals).

### Data collection

The questionnaire distributed to participants was a study-designed 13 item paper-based questionnaire adapted from the management competency studies conducted in Victoria, Australia in 2012 [27, 28]. The first study conducted in Australia was of a similar design, examining comparable managerial levels and different healthcare settings. The questionnaire was translated into Mandarin for this study. To minimise translation errors, the questionnaire was back translated to English by an independent translator and errors corrected. Questionnaire packages were distributed directly to and collected directly from the senior managers recruited to the study by one of the working group members. Each package contained project information, informed consent, the questionnaire and an opaque de-identified envelope to maintain anonymity. Participants were asked to complete the questionnaire independently and put the questionnaire into the enclosed stamped self-addressed envelope (regardless of whether it had been or had not been completed). To improve response rates, one of the project investigators provided a presentation to the Directors of 13 CHCs in the Fengtai District to brief them on the purpose of the research and procedure for participation. Directors then encouraged staff to participate in the study during their monthly management team meeting.

The items explored age, gender, years in current managerial role, years working for the organisation, highest qualifications obtained and commitment to formal education and training in the previous and in the coming year, and average length of their working hours per day and per week. Participants from the CHC were also asked to recall the total number of hours that they spent on continuous professional development, training organised internally and externally by the organisation in both management and non-management related areas. This question was only included in the survey with managers from CHCs as was specifically requested data by Directors of the CHCs.

Two of the 13 survey items included core tasks for managers to perform. For the identification of core tasks, a list of 12 managerial tasks were provided for managers to choose up to seven tasks that they believe were most important for them to perform as managers. The 12 tasks were adapted from the detailed job analysis conducted in Victoria, Australia, which were translated into Chinese language, discussed and finalised at a meeting with 20 health service managers from China. Additional space was provided for managers to provide details of additional managerial tasks that they believe are important but not included in the list.

### Data management and analysis

Data from the completed paper questionnaires were double entered into Microsoft Excel and checked for consistency and range. Descriptive analyses were completed in STATA version 13. The disciplines of qualifications and additional managerial tasks that managers believed were important to their management role were provided by managers. These qualitative data were grouped according to themes through content analysis.

Demographic, educational and work-related data were analysed by univariate analysis and by sector and management level. Data was analysed using standard frequencies and distributions. Descriptive and summary data used summary tables of participants within descriptive categories and used χ^2^ of descriptive variables/characteristics to consider ‘goodness of fit’ and if distribution was due to chance.

## Results

Overall, 94% of the managers from the 13 community health centres (268/283) and 80% of the hospital managers who received the questionnaire packages (231/260) returned the survey. Twenty-two questionnaires were excluded from the analysis, as managers who filled in these surveys did not indicate their management level and number of years in the management positions. Table 1 details the number of managers from each of the management levels from both CHC and hospitals whose returned questionnaire survey were included in the analysis and the respective response rate.

**Table 1:** Number of participants by management level and sector

Sector	Management Level	Number of participants	Response rate
**Community Health Centres**	**Level I**	27	87%
	**Level II**	105	95%
	**Level III**	136	96%
	**Total**	268	94%
**Hospitals**	**Level I**	17	85%
	**Level II**	84	84%
	**Level III**	108	77%
	**Total**	209	80%

Table 2 summarises the gender distribution by management level and sector. Compared to the hospital sector, a greater proportion of managers from CHC were female (78% vs. 54%) (χ^2^ = 30.5337, df = 1, *p* < 0.0005). In hospitals, 94% of the level I hospital managers were male. However, among level II and III managers the gender distribution was more equal (Level II, *F* = 52.5%; Level III, *F* = 62.3%). The distribution of gender in the CHC sector showed higher proportions of female managers across all levels of management, but the differences were not statistically significant (χ^2^ = 3.824, df = 2, *p* = 0.148).

**Table 2:** Gender of participants by sector and management level

	Community Health Centres			Hospitals		
	Level I	Level II	Level III	Level I	Level II	Level III
**Male**	37%	19%	22%	94%	49%	37%
**Female**	63%	81%	78%	6%	51%	63%

Table 3 details the age distribution amongst participants by management level and sector. Hospital managers were significantly older than managers working in the community health centres (χ^2^ = 34.45, df = 3, *p* < 0.0005). In CHC, Level I was the only management level with more than half of the participants aged 40 and above, whilst in hospitals, approximately 70% of the managers are aged 40 and above regardless of level. The distribution of ages within the hospital sector is statistically significantly different across the levels of management (χ^2^ = 13.62, df = 6, *p* = 0.034), but not statistically different in CHC (χ^2^ = 9.45, df = 6, *p* = 0.150).

**Table 3:** Age groups of participants by sector and management level

Age group (years.)	Community Health Centres			Hospitals		
	Level I	Level II	Level III	Level I	Level II	Level III
**< 30**	4%	8%	6%	7%	4%	9%
**30–39**	30%	49%	44%	22%	27%	20%
**40–49**	56%	28%	41%	43%	39%	47%
**> 50**	10%	15%	9%	28%	30%	23%

Table 4 summarises the highest qualifications possessed by participants from both sectors by management level. A significantly greater proportion of hospital managers have higher qualifications compared to managers working in community health services at all three management levels (χ^2^ = 193.96, df = 4, *p* < 0.0005). In hospitals, 93% of Level I, 72% hospital Level II and 75% Level III hospital managers possess either a Masters or Doctorate degree. In contrast, for managers from CHCs, the percentages for these levels are 28, 12 and 19% respectively.

**Table 4:** Highest qualifications of managers by sector and management level

	Community Health Centres			Hospitals		
	Level I	Level II	Level III (clinical)	Level I	Level II	Level III (clinical)
**TAFE/College**	6%	40%	20%	0	2%	0%
**Bachelor**	66%	48%	61%	7%	25%	25%
**Masters**	28%	12%	17%	36%	44%	30%
**Doctorate**	0%	0%	2%	57%	28%	45%
**Postgraduate Qualification**	28%	12%	19%	93%	72%	75%

Table 5 details the study disciplines of the highest qualifications possessed by participating managers. The highest qualifications possessed by 20% of the hospital Level I, 23% of the hospital level II and 27% of CHC Level I managers were management related. Only 2% of level III managers from either sector possessed a management-related highest qualification.

**Table 5:** Discipline of the highest qualifications possessed by sector and management level

	Community Health Centres			Hospitals		
	Level I (26)	Level II (95)	Level III (127)	Level I (10)	Level II (82)	Level III (83)
**Medicine**	42%	24%	38%	50%	44%	70%
**Nursing**	12%	28%	14%	0	20%	24%
**Management**	27%	9%	2%	20%	23%	2%
**Management related total**	8%			14%		

Thirty to 40 % of level I managers have recently completed or currently completing a degree course (Table 6). However, the majority of the Level II and Level III managers from both sectors have not planned to begin a degree course during the next year.

**Table 6:** Current commitment to or future plans for formal education by sector and management level

	Community Health Centres			Hospitals		
	Level I	Level II	Level III	Level I	Level II	Level III
**Nothing planned**	43%	68%	74.%	43%	70%	65%
**Starting within a year**	11%	14%	10.%	21%	15%	15%
**Currently studying**	28%	12%	9%	7%	6%	5%
**Just completed a degree**	18%	6%	7%	29%	9%	15%

Table 7 summarises the average hours for each management level for each type of training opportunity for CHC managers. The total hours spent on professional development and training each year is very similar between three management levels ranged from 154 and 176. Only 21–25% of such hours were spent on management related training (no specification of types of management training was provided). As mentioned in the method section, this question was only included in the survey with managers from CHCs as was specifically requested data by Directors of the CHCs.

**Table 7:** Average number of hours per year on professional development and training by management level at Community Health Centres

Training types		Level I	Level II	Level III
**Organised internally**	**Non-management related**	35	41	45
	**Management related**	13	20	21
**Organised externally**	**Non-management related**	41	45	48
	**Management related**	26	25	15
**Total hours/year**		154	176	165
**Total hours/year non-management specific training**		115	131	126
**Total hours/ year management related training**		39	45	36

Table 8 shows that hospital managers work longer hours per week than managers working at community health centres. More than half of the Level II and III hospital managers work more than 50 h per week while less than 10% of the community health managers at the same level are working this long. Further analyses of working hours per week shows that hospital managers tended to work longer hours per week than managers at CHC (χ^2^ = 130.18, df = 3, *p =* < 0.0005). Within sectors, there were no differences between levels of CHC managers (χ^2^ = 2.823, df = 6, *p* = 0.831). In the hospital sector, level II and III managers tended to work longer hours than level I and the differences were statistically significant (χ^2^ = 29.549, df = 6, *p =* < 0.0005).

**Table 8:** Average working hours per week by sector and management level

Hours per week	Community Health Centres			Hospitals		
	Level I	Level II	Level III	Level I	Level II	Level III
**30–39**	11%	12%	9%	12%	0	0
**40–49**	85%	80%	82%	53%	56%	39%
**50–59**	4%	7%	9%	35%	43%	45%
**> = 60**	0	0	0	0	12%	16%

The final question to be included in this paper was the managerial tasks that were considered important to managers working in the targeted community health centres and the level three teaching hospitals. Table 9 summarises the percentage of managers from each management level that have chosen the specific tasks as important to their role. Whilst there is considerable variation across sectors and management levels, some obvious similarities and differences stand out. Tasks 1, 2, 4, 6, 7 and 12 appear to be important across sectors and management levels, whereas tasks 10 and 11 are consistently rated of low importance. Overall, 69% of all tasks were considered important by 40% of managers or more. No additional tasks were identified by the participants.

**Table 9:** Tasks selected by percentage of managers by sector and management level

Tasks^b^		CHCs^a^			Hospital		
		LI	LII	LIII	LI	LII	LIII
**1**	Staff performance appraisal and professional development	47%	47%	49%	46%	68%	66%
**2**	Staff management and development	63%	57%	41%	62%	46%	54%
**3**	Performance and financial management	56%	46%	28%	38%	22%	47%
**4**	Provision of leadership to staff and key stakeholders	63%	57%	33%	72%	58%	33%
**5**	Revenue generation and resource allocation	44%	47%	47%	54%	32%	43%
**6**	Promotion and development of organisational image and public relations	67%	48%	54%	54%	49%	47%
**7**	Maintenance and improvement of quality and safety of service provision	81%	75%	76%	85%	68%	65%
**8**	Develop and oversee implementation of organisational strategic service	30%	47%	43%	8%	41%	50%
**9**	Development of organisational vision, strategic direction and policies	67%	34%	27%	62%	38%	46%
**10**	Oversee occupational health and safety and risk management in accordance with legal requirements	22%	34%	29%	15%	35%	42%
**11**	Develop and implement occupational health and safety and risk management	19%	26%	34%	28%	27%	30%
**12**	Effective information management to inform learning and development for staff and practice/service delivery	41%	54%	56%	46%	56%	55%

^a^ CHCs **=** Community Health Centres ^b^ Task are taken from Liang et al. (2018 )[29] as these are the tasks and competencies that have been validated for HSM

## Discussion

The results of this study indicate there are significant differences between those who are filling managerial roles in the public hospitals and the CHC organisations in China’s public healthcare system, suggesting that professional development needs for management competency of the two different sectors may be different. More specifically, gender distribution, highest qualifications, hours worked, years in the position and perceived managerial tasks appear to vary between the two different sectors. These will be explored in detail below.

### Age and gender

Managers working in the hospitals tend to be older than managers working in CHCs, which likely reflects the fact that majority of the community health centres in China were established in the 1990s and are a younger model of healthcare delivery. In China, it appears that managers are generally promoted internally in the organisation with an age limit, and most managers stay with the same organisation throughout their career, which may explain why the majority of the managers working in both sectors are within the age of 50. It is worth noting that this finding is comparable to our study conducted in Victoria, Australia, where the mean age of middle to senior level managers working in the community health services and public hospitals was 49.5 years [30].

There are gender imbalances in health services managers in China, with the CHC sector seeing more females, and upper level management in the hospital sector being predominately males. Our study in Australia also found that about 70% of the senior and middle level managers working in both community health services and public hospitals were female. The gender imbalance swinging towards more males amongst Level I managers working in the public hospital in China is surprising as this phenomenon has not been identified through our literature review at the beginning of the study nor was observed in Australia. Further research to appreciate the reasons for the significant gender imbalance is needed to be able to then ensure that senior management in Chinese hospitals has an appropriate distribution of male and female workforce.

### Qualifications of managers

There are significant differences in the qualifications possessed by managers working in CHS and level III hospitals, and between management levels within the same sector. More than three quarters of level I to level III managers have obtained a Master or Doctorate level qualifications in the hospital sector. In contrast, less than 20% of the Level I to Level III managers possessed postgraduate qualifications in the CHC sector. In China, University affiliating hospitals are very large institutions that provide the most complex and high level medical treatments to patients come from all over the Province with teaching and research capacity. As mentioned in the background section, management positions are normally recruited internally based on clinical seniority. Vast majority of the management positions carry dual role without giving up clinical practice. Hence, it is a common phenomenon that clinicians are recruited to management positions without management qualification requirements. Committing to clinically based postgraduate qualifications are more beneficial to their both clinical and management career advancement. On the other hand, CHCs are very small institutions and only provide outpatient services and other types of community-based service such as health promotion and early intervention. Management position is less demanding and competitive. While the differences observed across managerial levels is to be anticipated, as different levels require different levels of capability, it is unclear as to why there are significant differences between the CHC and hospital sectors at each level. To date, no research has explored the reasons for the existence of the differences. Historically, primary care institutions in China were staffed by those who do not have a university degree (the legacy of barefoot doctors). This has gradually been phased out. In Beijing and other developed regions, CHCs may be able to attract those with higher degrees from other less-developed regions. Although, based on the working knowledge and understanding of the educational system in China, this difference may be explained by two factors.

Firstly, Masters level qualifications in China are primarily research-based and offered on a fulltime basis, except for the small number of MBA and EMBA programs. Managers working in the CHC may find participation in these programs a challenge as it is highly likely they would be required to take leave of absence from their positions due to the fulltime demands of study, and the geographical location of the Universities that offer postgraduate study being different from the workplace. Unlike hospitals, CHCs are not specifically affiliated with universities in China and therefore, lack the opportunity to collaborate with research projects supporting enrolment in a Masters by research degree. This compares with managers at Level III public hospitals affiliated with a medical university where there are opportunities for managers to pursue their study while working full or part time. Future research exploring the perceptions of CHC managers to undertake postgraduate qualifications using qualitative research methods is recommended to develop an understanding of the barriers and facilitators for completion of postgraduate learning in China.

Secondly, apart from a small number of MBA and EMBA programs, all potential postgraduate candidates are required to take part in the standardised National Postgraduate Entrance Examination (NPEE) as part of the admission requirement for all graduate schools in mainland China [31, 32]. This examination also includes College-English Test 6. Although no actual passing rate of NPEE is available, the opportunities for admission to postgraduate study in mainland China are competitive and enrolments are limited. International opportunities to study again provide the barrier of requiring the person to take a leave of absence from their paid employment, and there is potentially a significant financial burden with tuition fees and overseas living expenses. Equally challenging for postgraduate studies overseas is the pre-requisite language requirements of the host educational organisation. Managers in China may not be eligible for enrolment in the programs offered in English (or other language) speaking countries.

The recent study on management competency in Australia conducted by the authors of this paper confirmed that approximate 64% of the senior managers working in both the public hospitals and community health services in Victoria (one of the largest Australian States) possessed a postgraduate qualification [30]. The study found no significant differences in the postgraduate qualification possessed by managers between community health services and hospitals and between the three senior management levels. Amongst the postgraduate qualifications that Australian health service managers possessed, one third of them are management related. This is a significant contrast to the types of postgraduate qualifications possessed by Chinese managers, as identified in this study, where less than 15% of qualifications are management related. The results of this study suggest that advance of a persons’ management career is still vastly based on seniority in clinical practice, the motivation behind developing management competence prior to and after taken up management roles are lacking. Management specific formal training does not yet appear to be one of the requirements specified in position descriptions of manager in the healthcare sector in China. The reasons may be the lack of recognition of formal management training; lack of practical and course-based management degrees offered in Chinese Universities and the difficulties for managers taking time off to commit to fulltime study.

Formal training and education in health service management / health administration has received greater support among developed countries [5, 33]. Training managers effectively is regarded as an important HRM practice for improving the competence of health service management workforce [34, 35]. This is partly due to the generation of evidence linking the competence of health managers with better health service delivery and linking the improved competence of health managers via formal management training and education [33, 36, 37]. However, given the extensive reliance of the Chinese healthcare system on the internal management promotional pathway, and the lack of training and preparation provided to health professionals prior to incumbency of management positions, the adoption of formal management training is both challenging and possibly even unlikely. This is also exacerbated by the lack of importance and availability of informal management training to health service managers in the Chinese Healthcare sector. Instead, we suggest that informal education together with coaching and mentoring may provide an alternative way of improving the overall competence of the Chinese health service managers. Establishment and/or empowerment of health administration professional associations could help to address this need for informal training. This would ensure that the professional development opportunities reflect best practice, avoiding the need to rely on in-house training exercises that may perpetuate mistakes made through a lack of insight into new approaches. In addition, mentoring and coaching requires a system to connect mentors and mentees together, and a professional association would support this. Sharing of lessons learnt between mentor/mentee across the country and globally could underpin transformation of practice in new ways. Research exploring the career pathways of senior healthcare managers and the process by which management training and support for professional development can be successfully implemented is necessary to determine the most effective and efficient next steps.

### Commitment to continued professional development and continuous education of senior managers

Our study shows that more than a third of the Level I managers working in public hospitals and nearly half of the Level I managers working at CHC have recently completed, or are currently completing, a degree course. However, the clear majority of the Level II and III managers working in CHC and hospitals do not have any plans for any further formal education. Level II and III managers normally are those responsible for the service delivery of the division of which they are in charge. Therefore, their managerial competence will have significant impact on the effectiveness and efficiency of the health service delivery. Although the majority of the Level II and III CHC managers do not have plans for formal education, all three management levels spent similar amount of time (ranging between 131 and 115 h per year) participating in the informal training organised internally and externally by the organisation, usually in the form of seminars at the workplace. This suggests that to improve competency, the most desired process would be through informal courses organised internally or externally to the organisation, rather than enrolled degrees at University. This aligns with the early point that managers may find it a challenge to be absent from work for formal study. Chinese managers appear to invest time in participating in management related training when training does not require their absence from the paid position for a lengthy period. This suggested reasoning is important considering that senior managers in the Chinese health system need to obtain approval from the governments to participate in formal and informal training, which may not always be an easy process [25].

Approximately 30% of the participated informal training is management related, with the remaining 70% of training in clinical practice. This is a significant finding for the development of competency in managerial roles. Despite the existence of policy requirements of management training, this finding suggests that limited attention is devoted to learning about managerial positions and effective and efficient healthcare service leadership although there has been a policy in strengthening the management workforce by the participation of relevant management training. Research to understand why professional development in managerial skills is not being undertaken is required, followed by a review of the impact of a lack of managerial competency on healthcare provision, in terms of both patient care and economic outcomes. Such research will then underpin the next steps in designing professional development opportunities. Where access and time is determined to be of concern, online learning opportunities or short courses may overcome barriers. Where credentialled learning is a key motivator for engaging in professional development, formal courses may be more applicable.

### Core responsibilities for senior managers working in the community health centres and hospitals

Based on the experiences working with Chinese health service managers, it is not surprising to note that managers working in the Chinese health system are not provided with a job description specifying specific management responsibilities. Managers respond to high-level orders and the demand from running the departments for which they are responsible [26]. The literature on studies conducted on health service management in China is very limited and this is the first study to identify perceived responsibilities of Chinese health services managers. There appears to be responsibilities that are common to all management levels across both CHC and the public hospital system, and responsibilities that are unique to different management levels and sectors. This may directly relate to differences in patient population, as well as the different missions and functions of the differing settings.

According to the pyramidal relationship between tasks, roles and competencies [27, 28], identification of the core tasks that health managers spend most of their working hours is the first step in confirming the most important competencies (knowledge, skills and attitudes) required for managers to fulfil their managerial role effectively. The confirmation of the tasks in this study provides very useful information for preparing potential managers in taking up management roles and to developing better understanding of the management competency requirements. It can also be used as a foundation for design educational activities to support professional development, by identifying perceived learning needs to be a competent manager.

The two tasks that are seen as essential across all management levels in both sectors are:

- Promotion and development of organisational image and public relations, and
- Maintenance and improvement of quality and safety of service provision.

High profile adverse events and conflicts between doctors and patients have been the centre of attention and published widely in the media lately in China [38]. These reports also include violence toward clinicians. This reflects the urgency for health care organisations to build a positive image as providers of safe and quality healthcare services, of which highly competent and skilled managers are crucial.

The international literature suggests leadership is a core competency for managers and all health professionals [27, 39]. Our research in Australia confirmed leadership as one of the key competencies for managers across sectors and management levels [27, 28]. However, the importance of leadership was well recognised by level I and II hospital managers and CHC Level I and II managers, but not hospital and CHC Level III managers. Two reasons may lead to such lack of recognition. First, this study shows that CHS Level II and III managers have less opportunity to be exposed to formal and informal management training, which may not allow them to develop the awareness of such competencies. Second, as mentioned earlier, managers are promoted within the organisation and by higher order. CHCs are much smaller in size than hospitals; therefore, managers from the functional and clinical department / division may not carry the same level of leadership responsibilities as of the senior managers in the hospitals and Directors of CHC. The lack of autonomy in managing the department and division also attributed to the lack of awareness of the importance of leadership. In future planning of educational activities for development of management competency in China, a focus on leadership development is likely to be highly regarded.

### Strengths and weaknesses

The major strength of the study is the sample size and high response rates across sectors and management levels. One of the weaknesses of the study was the reliance on an accurate translation of the questionnaire from English to Mandarin. Although steps were taken to minimise mistranslation, some random misunderstanding of the questions by the participants was unavoidable. Another problem experienced while writing the paper was the transient nature some of the references of the data sources due to frequent changes to Chinese government websites. Where possible, only permanent publications have used. As this is a cross sectional survey, the data captured is also at a single point in time, and potentially may change over time. Finally, the study was conducted in a specific region of China, which limits generalizability of results. Cultural and geographical factors may influence access to managerial training and shape the mission and function of the healthcare settings which in turn drives who the health services managers are and what capabilities they have or need to development. None-the-less, this is the first study exploring this important topic, and insights into future directions will support new research in the area.

## Conclusions

This study determined that there are differences between the demographics of managers in China across levels of management, but more importantly between the CHC and the hospital sectors. The historical development and policy of management career advancement within the healthcare system may partially explain such differences, and warrants further research to explore the underlying reasons. More significantly, the study strongly argues the importance of informal management training, coaching and mentoring for developing the health service management workforce. Research is needed to understand ways of incorporating such training and support in at the various career stages of health professionals who may eventually become health managers in the health system. Research is also needed to appreciate the capability needs to allow targeted training and maximise healthcare service provision in China. This paper gives insights into potential professional development opportunities for health services managers in China, including the desire for informal education and focusing on the core roles of promotion and development of organisational image and public relations, improvement of quality and safety of service, and the provision of leadership. Investment in the capability development of the health services managers in China could lead to improved service provision with greater economic sustainability and improved public health outcomes.

## Data Availability

The datasets used and/or analysed during the current study are available on reasonable request and with permission of the corresponding author.

## Abbreviations

*CHC*: Community Health Centre
*EMBA*: Executive Masters of Business Administration
*HRM*: Human resource management.
*MBA*: Masters of Business Administration
*NPEE*: National Postgraduate Entrance Examination
*UK*: United Kingdom
*US*: United States
